# LRRK2 Kinase Inhibition Rescues Deficits in Lysosome Function Due to Heterozygous *GBA1* Expression in Human iPSC-Derived Neurons

**DOI:** 10.3389/fnins.2020.00442

**Published:** 2020-05-15

**Authors:** Anwesha Sanyal, Hailey S. Novis, Emile Gasser, Steven Lin, Matthew J. LaVoie

**Affiliations:** Ann Romney Center for Neurological Diseases, Department of Neurology, Brigham and Women’s Hospital, Harvard Medical School, Boston, MA, United States

**Keywords:** LRRK2 kinase inhibition, GBA1 deficiency, lysosomal dysfunction, cellular trafficking, iPSC-derived neuron

## Abstract

A growing number of genes associated with Parkinson’s disease are implicated in the regulation of lysosome function, including *LRRK2*, whose missense mutations are perhaps the most common monogenic cause of this neurodegenerative disease. These mutations are collectively thought to introduce a pathologic increase in LRRK2 kinase activity, which is currently a major target for therapeutic intervention. Heterozygous carriers of many missense mutations in the *GBA1* gene have dramatically increased risk of Parkinson’s disease. A critical question has recently emerged regarding the potential interplay between the proteins encoded by these two disease-linked genes. Our group has recently demonstrated that knockin mutation of a Parkinson’s-linked *GBA1* variant induces severe lysosomal and cytokine abnormalities in murine astrocytes and that these deficits were normalized via inhibition of wild-type LRRK2 kinase activity in these cells. Another group independently found that LRRK2 inhibition increases glucocerebrosidase activity in wild-type human iPSC-derived neurons, as well as those whose activity is disrupted by *GBA1* or *LRRK2* mutation. Fundamental questions remain in terms of the lysosomal abnormalities and the effects of LRRK2 kinase inhibition in human neurons deficient in glucocerebrosidase activity. Here, we further elucidate the physiological crosstalk between LRRK2 signaling and glucocerebrosidase activity in human iPSC-derived neurons. Our studies show that the allelic loss of *GBA1* manifests broad defects in lysosomal morphology and function. Furthermore, our data show an increase in both the accumulation and secretion of oligomeric α-synuclein protein in these *GBA1*-heterozygous-null neurons, compared to isogenic controls. Consistent with recent findings in murine astrocytes, we observed that multiple indices of lysosomal dysfunction in *GBA1*-deficient human neurons were normalized by LRRK2 kinase inhibition, while some defects were preserved. Our findings demonstrate a selective but functional intersection between glucocerebrosidase dysfunction and LRRK2 signaling in the cell and may have implications in the pathogenesis and treatment of Parkinson’s disease.

## Introduction

LRRK2 is a large multi-domain protein that functions both as a kinase and a GTPase (West et al., 2005; Gloeckner et al., 2006; Biosa et al., 2013; Nguyen and Moore, 2017). Autosomal dominant missense mutations in *LRRK2* are causative for familial PD and further linked to sporadic forms of the disease (Van Den Eeden et al., 2003; von Campenhausen et al., 2005; Healy et al., 2008; Gasser, 2009; Kalia et al., 2015). LRRK2 is expressed in various organs including brain, lung, kidney and circulating immune cells and its function has been implicated in several cellular signaling pathways including cytoskeletal polymerization, vesicular trafficking, synaptic transmission, mitochondrial function and regulation of the autophagy-lysosomal system (Inestrosa and Arenas, 2010; Papkovskaia et al., 2012; Migheli et al., 2013; Schapansky et al., 2014; Cookson, 2015; Taymans et al., 2015). Studies in aged *LRRK2* knockout rodents and those involving reductions in LRRK2 activity by knockdown or pharmacological interventions have indicated an important role of LRRK2 in maintaining proper lysosomal function (Tong et al., 2010; Herzig et al., 2011; Hinkle et al., 2012).

The pathology observed in LRRK2-PD most commonly includes the age-dependent accumulation of insoluble α-synuclein (αSyn) and classic neuronal Lewy body formation (Alegre-Abarrategui et al., 2008; Vitte et al., 2010; Yacoubian et al., 2010). αSyn can be degraded both by the proteasome and the lysosome and its deposition in PD could conceivably arise from deficits in either pathway (Webb et al., 2003; Yan et al., 2018). Inhibition of autophagy or endo-lysosomal function leads to an accumulation of αSyn, indicating the importance of this pathway in αSyn degradation (Zimprich et al., 2004; Fornai et al., 2005). Furthermore, αSyn proteostasis is fundamentally linked to LRRK2 activity (Cuervo et al., 2004; Fornai et al., 2005; Schapansky et al., 2018). Accumulation of αSyn is observed in *LRRK2* knockout rodent kidneys, LRRK2 G2019S knock-in mouse neurons, and LRRK2 G2019S iPSC-derived dopaminergic neurons (Hernandez et al., 2016; Pellegrini et al., 2018; Bieri et al., 2019). Thus, there is an established causal link between altered LRRK2 activity and αSyn metabolism, likely involving dysfunction of the endo-lysosomal system.

A wide series of Rabs, members of a protein family critical to intracellular transport across the endo-lysosomal system and beyond, have been determined to be phosphorylated by LRRK2 (Steger et al., 2016). This observation likely explains the complicated lysosomal phenotypes associated with increased or defective LRRK2 kinase activity in cells (Tong et al., 2010; Hockey et al., 2015; Schapansky et al., 2018). New questions are emerging with respect to the impact of LRRK2 signaling under conditions where endo-lysosomal trafficking is perturbed by stressors other than LRRK2 mutation, and how modulation of LRRK2 activity would impinge upon such environments. Autosomal recessive mutations in *GBA1*, which codes for the enzyme glucocerebrosidase (GCase), are causal for the lysosomal storage disorder Gaucher’s disease, whereas heterozygous carriers are at significantly greater risk of PD (Neumann et al., 2009; Sidransky et al., 2009; Bultron et al., 2010; McNeill et al., 2012). We recently showed that a loss-of-function mutation in *GBA1* leads to lysosomal defects in murine astrocytes that could be normalized by inhibition of LRRK2 kinase activity (Sanyal et al., 2020). Excess LRRK2 kinase activity has also been shown to negatively regulate GCase activity in dopaminergic neurons, likewise corrected with LRRK2 inhibitors (Ysselstein et al., 2019). Taken together, these observations suggest a physiological link between LRRK2 and GCase in a convergent signaling pathway that exists across multiple cell types. Given the clear impact of these mutations on the lysosome, we sought greater insight into the status of LRRK2 signaling in *GBA1*-deficient human iPSC-derived neurons and how LRRK2 kinase inhibition would affect *GBA1*-dependent phenotypes.

Recent advances in iPSC technology allowed us to generate a series of WT and isogenic CRISPR/Cas9-engineered heterozygous-null *GBA1* human iPSCs. Differentiating these cells into cortical layer 2/3 induced neurons (iNs) offers us the unique opportunity to examine PD-relevant phenotypes in heterozygous-null *GBA1*-mutant human neurons. In this study, we found that heterozygous-null *GBA1* iNs exhibit broad lysosomal defects. Specifically, we found decreases in lysosome number, increases in lysosomal pH, and reductions in lysosomal cathepsin protease activities. We then assessed whether these changes were sufficient to adversely affect αSyn metabolism in neurons. We observed an increased accumulation of soluble and insoluble αSyn without corresponding changes in αSyn mRNA levels, characteristic of αSyn dyshomeostasis. Furthermore, results showed an increase in the secretion of oligomeric αSyn. Next, we assessed endogenous LRRK2 activity and found that *GBA1* heterozygosity did not affect WT LRRK2 kinase activity. However, given the overlap between GBA1 and LRRK2 signaling reported in recent studies, we assessed the effects of LRRK2 kinase inhibition on *GBA1*-deficient neurons and found that pharmacological kinase inhibition of LRRK2 rescued selective lysosomal deficits, while not impacting others. This study confirms a broad physiological cross-talk between the cellular consequences of GCase dysfunction and the signaling of WT LRRK2, extending these data to now include both neurons and non-neuronal cells. The common pathways defined by these studies may deepen our understanding of PD etiology, as well as provide new opportunities to intervene in pathogenic processes in a therapeutic manner.

## Materials and Methods

### CRISPR/Cas9 Genome Editing of Human iPSCs

Single guide RNAs (sgRNA) for *GBA1* knockout (forward 5′-CACCGTTGGCTCAAGACCAATGGAG-3′ and reverse 5′-AAACCTCCATTGGTCTTGAGCCCAC-3′) were selected using a web-based design tool^1^. This was then cloned into pXPR_003 (Addgene #52963), that was modified to express the neomycin resistance gene instead of the puromycin resistance gene. Plasmid DNA was then commercially sequenced using the primer 5′-GATACAAGGCTGTTAGAGAGATAATT-3′ to determine clones that successfully integrated the sgRNA. BR01 and BR33 iPSCs were generated and characterized in collaboration with the New York Stem Cell Foundation (NYSCF) using previously described methods (Paull et al., 2015; Muratore et al., 2017). BR01 and BR33 were derived from a Caucasian female and male donor respectively, who were deeply phenotyped as part of the ROS/MAP longitudinal aging studies and determined to not be cognitively impaired at death at age >89 and free from genetic variants that confer risk of PD (Bennett et al., 2018). iPSCs were co-transfected with plasmids that express dCas9 (Addgene 61425) and the sgRNA plasmid. One microgram of each plasmid was transfected using 6 μL of Lipofectamine 2000 (Invitrogen) into a well of 90% confluent iPSC cells. After 2 days, cells that were successfully transfected with the two plasmids were selected by puromycin treatment for 4 days. Polyclonal cells were then monoclonally selected by plating ∼1 cell per well in a 96-well dish and allowed to grow for 2 weeks. Monoclonal lines were then expanded, sequenced and stocked. Amplification of *GBA1* gene was conducted with primers specifically designed to exclude *GBA1* pseudogene (forward 5′-CAGAAAGGCCTGCGCTTCA-3′ and reverse 5′-AAGGCTGAAAGGCCCAGAAG-3′), which was TA cloned according to manufacturer’s protocol (Invitrogen K2030-01) and heterozygous gene editing was confirmed by Sanger sequencing.

### Differentiation of Human iPSCs

iPSCs were cultured in StemFlex (Life Technologies A33493) media. 100,000 cells/cm^3^ were co-transduced with lentivirus packaged with pTet-O-NGN2-puro and Fudelta GW-rtTA plasmids (Zhang et al., 2013) for 2 days and passaged for expansion and frozen as stocks. NGN2-transduced iPSC cells were thawed in StemFlex media with ROCK inhibitor (10 μM; Stemcell Technologies, 72304) and plated at 2 × 10^6^ cells/10 cm plate and grown until 75% confluent. For differentiation, on day 1 these cells were fed with KnockOut media (Gibco 10829.018) supplemented with KnockOut Serum Replacement (Invitrogen 10928-028), 1% MEM non-essential amino acids (Invitrogen 11140), 1% GlutaMAX (Gibco 35050061) and 0.1% BME (Invitrogen 21985-023) (KSR) with doxycycline (2 μg/ml, Sigma, D9891-5g) to induce NGN2 expression. On day 2, they were fed with a 1:1 ratio of KSR:N2B media (DMEM F12 supplemented with 1% GlutaMAX, 3% dextrose and N2-Supplement B; StemCell Technologies 07156) with puromycin (5 μg/ml; Life Technologies, A11138-03) and doxycycline to select for transduced cells. On day 3, the cells were fed with N2B media with B27 (1:100; Life technologies, 17504-044), puromycin, and doxycycline. On day 4, induced neurons (iNs) were frozen down in 10% DMSO/FBS in Neurobasal media (NBM Gibco 21103-049) supplemented with B27, BDNF (Peprotech, 450-02), CNTF (Peprotech, 450-13), and GDNF (Peprotech, 450-10) all at 100 ng/uL, ROCK inhibitor (10 μM), puromycin, and doxycycline. iNs were plated and grown in NBM with B27, BDNF, CNTF, GDNF, puromycin, and doxycycline until day 21. All treatments were carried out at day 18–21.

### Western Blot

Cells were lysed in cell lysis buffer (50 mM Tris-HCl, 150 mM NaCl, 0.5 mM EDTA, 0.5% (v/v) sodium deoxycholate, 1% (v/v) NP-40, pH 8) with protease and phosphatase inhibitor for 30 min. The lysates were centrifuged at 14,000 × *g* for 15 min at 4°C, and the supernatant protein was quantified using BCA assay. Total protein was normalized, mixed with 1x SDS-PAGE loading buffer, denatured at 95°C for 5 min and resolved on an SDS-polyacrylamide gel, and transferred to PVDF membrane. For dot blots, conditioned media was blotted on a nitrocellulose membrane without boiling. Membranes were blocked with 5% bovine serum albumin (Sigma). Blots were probed with primary antibodies to LAMP1 (abcam ab108597), GCase (abcam ab55080), pT73-Rab10 (abcam ab230261), Rab10 (cell signaling 8127S), pT72-Rab8a (abcam ab230260), Rab8a (abcam ab188574), pS935-LRRK2 (abcam 133450), LRRK2 (clone, 8629). αSyn oligomer (abcam ab209538). Secondary antibodies conjugated to horseradish peroxidase were used for detection using autoradiography.

### Immunofluorescence

Cells were washed with PBS, fixed with 4% (w/v) paraformaldehyde, blocked with 5% (v/v) BSA in PBS for 30 min, and permeabilized with 0.1% (v/v) Triton X-100 in PBS for 5 min or digitonin for 30 min. Primary antibody to NGN2 (Abnova H00063973-M10), NeuN (Millipore MAB377), MAP2 (abcam ab32454) and αSyn (clone 15G7, Enzo Life Sciences ALX-804-258-LC05) was incubated for 1 h and washed with PBS; the secondary antibody conjugated to Alexa Fluor dye was incubated for 1 h, washed with PBS, and visualized by confocal microscopy (Zeiss LSM710).

### Glucocerebrosidase Activity Assay

Cells were resuspended in homogenization buffer (250 mM Sucrose, 10 mM Tris pH 7.5, 1 mM EDTA, 025% Triton X-100) and the cell pellet was disrupted on ice with a probe sonicator thrice for 5 s at 50 W. The cell lysate was centrifuged at 20,000 g for 20 min at 4°C, protein concentration quantified and normalized to 1 μg/μl. In a 96-well plate, 25 μl/well cell lysate, 100 μl of assay buffer (0.2 M sodium phosphate dibasic, 0.1 M citric acid), 25 μl of 4-methylumbelliferyl β-D-glucopyranoside substrate, incubated for 30 min at 37°C, reaction stopped with 75 μl stop solution (1 M Glycine, pH 10.5) and fluorescence read at 355 nm excitation 450 emission.

### Neurite Outgrowth Assay

Live-cell imaging using the IncuCyte ZOOM live imaging system (Essen BioScience) was started immediately after plating iNs after differentiation at DIV 5 in 96 well plate, assigning 4 fields per well, 6 wells per genotype for each of three independent differentiations. Neurite length and neurite branch point were measured using the Essen BioScience neurite analysis tool after imaging every 4 h for 3 days.

### High Content Analysis of Lysosomal Morphology

Neurons were plated 10,000 cells per well in 96-well black-wall clear-bottom plates (Greiner), labeled with LysoTracker^®^ Red (Invitrogen) according to the manufacturer’s specifications, and 20 ng/ml of Hoechst. Labeled live cells were imaged at 10x magnification, six fields per well, in the DAPI and Cy3 channels using an IN Cell Analyzer 2200 (GE Healthcare). Images were analyzed with the IN Cell Workstation software (GE Healthcare) multi target analysis protocol. Briefly, nuclei were segmented by applying a Top Hat algorithm with a minimum area of 50 square μm and a sensitivity level of 50 to the DAPI channel. Lysosomes were defined as objects with a 1–3 μm diameter, segmented by 2 scales with a sensitivity level of 20 in the corresponding channel. Cell count, lysosome count, mean lysosome area and total lysosome area were calculated.

### LysoSensor Assay

For lysosomal pH analysis, the ratiometric dye LysoSensor^TM^ Yellow/Blue (Invitrogen) was used. Neurons were plated 10,000 cells per well on 96-well black-wall black-bottom plates (Thermo Scientific), labeled with dye (1 μM) for 10 min prior to rinsing 2x with HBSS buffer. Cells were imaged using a Synergy H1 hybrid reader (Bio-Tek; reading at excitation 329/384, emission 440/540). Then, cells were incubated for 5 min at 37°C with pH calibration standards (pH of 3.96, 4.46, 4.96, 5.47, and 5.97) prepared in 20 mM 2-(N-morpholino)ethanesulfonic acid, 110 mM KCl, and 20 mM NaCl freshly supplemented with 30 μM nigericin and 15 μM monensin. A pH standard curve was determined for each genotype using GraphPad Prism 7 and individual baseline pH values were interpolated from these standard curves.

### DQ-BSA Assay

For lysosomal protease activity analysis, DQ-BSA^TM^ conjugate dye (Life Technologies) was used. Neurons were plated 10,000 cells per well in 96-well black-wall clear-bottom plates (Greiner), labeled with dye (1 μM) for 10 min and 20 ng/ml of Hoechst prior to rinsing 2x with HBSS buffer Total fluorescence intensity per well was quantified using a Synergy H1 hybrid reader (excitation 505 nm, emission 515 nm). For normalization, Hoechst staining is quantified (excitation 365 nm, emission 480 nm). For representative images, cells were imaged at 10x magnification, six fields per well, in the DAPI and GFP channels using an IN Cell Analyzer 2200 (GE Healthcare).

### Cathepsin Activity Assays

Neurons were plated 10,000 cells per well in 96-well black-wall clear-bottom plates (Greiner), labeled with 1 μM Magic-Red^TM^ dye (Bio-Rad, ICT937 and ICT 941) for 10 min and 20 ng/ml of Hoechst prior to rinsing 2x with HBSS buffer. Total fluorescence intensity per well was quantified using a Synergy H1 hybrid reader (excitation 592 nm, emission 628 nm). For normalization, Hoechst staining is quantified (excitation 365 nm, emission 480 nm). For representative images, cells were imaged at 10x magnification, six fields per well, in the DAPI and Texas-red channels using an IN Cell Analyzer 2200 (GE Healthcare).

### RNA Isolation, RT, and qRT-PCR

Total cell RNA was extracted with the RNeasy mini plus kit (Qiagen). Five μg of RNA was reverse transcribed with random primers using the Superscript IV First Strand Synthesis System (Life Technologies). Two μl of a 1:10 dilution of cDNA was used for quantitative PCR with gene specific primers and SYBR Green PCR master mix (Applied Biosystems A25742) according to manufacturer’s instructions. Human gene-specific primer sequences were as follows: GBA1 (forward 5′-CTCCATCCGCACCTACACC-3′ and reverse 5′-ATCAGGGGTATCTTGAGCTTGG-3′), αSyn (forward 5′-CTGCTGCTGAGAAAACCA-3′ and reverse 5′-CCT TGGTTTTGGAGCCTA-3′) and Actin (forward 5′-ATTGCC GACAGGATGCAG A-3′ and reverse 5′-GAGTACTTG CGCTCAGGAGGA-3′)

### Statistical Analyses

All experiments were conducted at least three independent times for three differentiations. Error bars indicate mean +SEM. Statistical analysis was performed using GraphPad Prism software, using a one-way ANOVA with Tukey’s *post-hoc* test.

## Results

### *GBA1* Heterozygous-Null iPSCs and iNs Exhibit a Gene-Dose Dependent Decrease in GCase Protein and Activity

**FIGURE 1:**
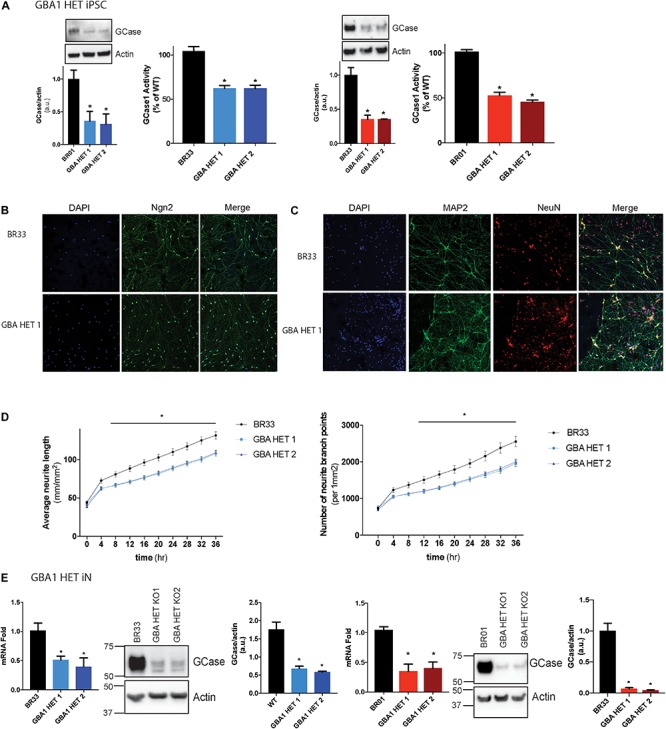
*GBA1* heterozygous-null iPSCs and iNs. **(A)** Western blot analysis of GCase and actin proteins in WT and *GBA1* heterozygous-null clones in two independent iPSC backgrounds, BR01 and BR33. GCase activity from protein normalized to whole-cell lysates using a GCase-specific fluorogenic substrate. **(B)** WT and *GBA1* heterozygous-null induced neurons (iNs) were analyzed by immunofluorescence assay for neuronal markers NGN2 (green), **(C)** NeuN (green) and MAP2 (red). Nuclei were counterstained with DAPI (blue). **(D)** Average neurite length and neurite branch points were quantified every 4 h over 3 days in WT and *GBA1* heterozygous-null iNs. Data was collated from 6 wells for each of three differentiations (*N* = 3, *p < 0.01, 2-way repeated measures ANOVA followed by Tukey’s *post-hoc* test). **(E)** Quantitative PCR analysis of *GBA1* mRNA in WT and *GBA1* heterozygous-null iNs in the BR01 and BR33 background. Western blot analysis of GCase and actin protein levels in *GBA1* heterozygous iNs in all the clones. All analyses were collated from three independent experiments for each of three differentiations on 21-day old iNs (*N* = 3, *p* < 0.001).

Here we used CRISPR/Cas9 based genome editing technology to create isogenic clones of *GBA1* heterozygous-null human iPSCs in two independent wildtype healthy control iPSC lines (BR01 and BR33). These cells were first tested for the loss of GCase protein and two clones for each WT iPSC background were chosen for further studies. We observed a 50–70% loss of protein in each clone (Figure 1A). In addition, we confirmed ∼50% corresponding loss of GCase activity (Figure 1A). Sanger sequencing indicates an insertion at 584 bp (GBA1/BR01 Het 1), a premature stop at 589 bp (GBA1/BR01 Het 2), insertions at 584 and 675 bp (GBA1/BR33 Het 1) and a frameshift from 592 bp (GBA1/BR33 Het 2) (Supplementary Figure S1A). Next, we differentiated these cells to “induced” layer 2/3 cortical neurons (iNs) via the forced expression of NGN2 (Zhang et al., 2013), Immunofluorescence staining of NGN2 confirms the high efficiency of transduction and robust expression of NGN2 in WT and *GBA1* heterozygous-null neurons (Figure 1B). In addition, staining with neuronal markers NeuN (which localizes to the nucleus) and MAP2 (which localize to the cell body and neurites) revealed structural integrity of the neurons and confirms equal efficiency of differentiation across different genotypes (Figure 1C). Interestingly, while not apparent by eye, when we quantified the outgrowth of neurites over 3 days, we found a subtle but consistent and significant decrease in average neurite length and neurite branch points in the *GBA1* heterozygous-null iNs when compared to their isogenic WT neurons (Figure 1D). Importantly, these iNs maintained the reduction in GCase protein and mRNA after differentiation and maturation for 21 days in culture (Figure 1E). Thus, we successfully generated a human neuronal model that recapitulates partial loss of GCase function in isogenic heterozygous-null *GBA1* neurons, a critical aspect of GBA1-linked PD.

### Broad Lysosomal Impairment Is Observed in *GBA1* Heterozygous-Null Neurons

**FIGURE 2:**
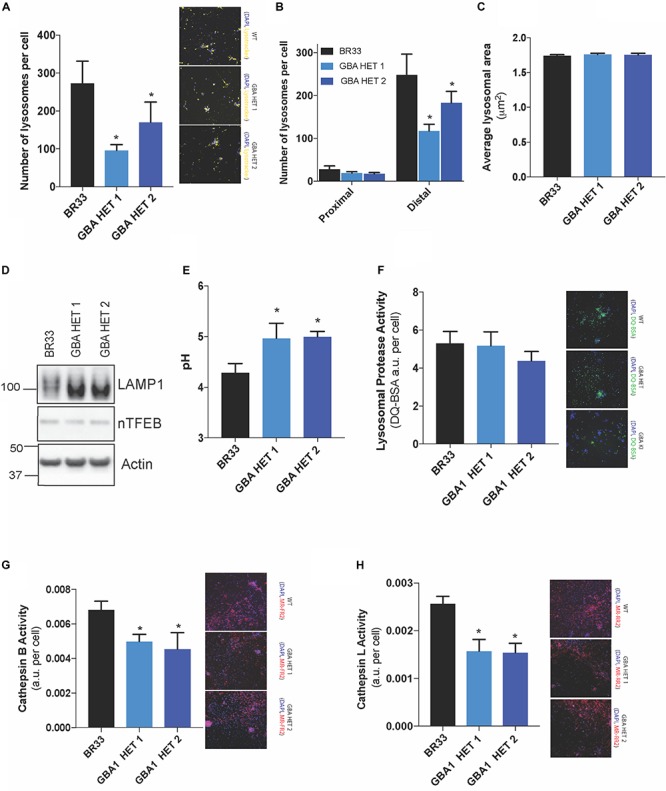
Broad lysosomal impairment is observed in *GBA1* heterozygous-null neurons. High-content image analysis, as detected by Lysotracker^TM^ staining normalized to number of cells, show **(A)** lysosome number in WT and two clones of *GBA1* heterozygous-null iNs, **(B)** lysosomal number in cell bodies (proximal) vs. neurites (distal) and **(C)** average lysosomal area in these cells. Cy3 fluorescence from Lysotracker^TM^ staining is observed in representative microscopy images. **(D)** Western blot analysis of LAMP1, nuclear TFEB, and actin proteins in WT and *GBA1* heterozygous iNs. **(E)** Determination of lysosomal pH using LysoSensor^TM^ in WT and *GBA1* heterozygous iNs. **(F)** General lysosomal protease activity, as detected by DQ-BSA cleavage (green), in WT and *GBA1* heterozygous iNs. Green fluorescence due to cleavage of DQ-BSA is observed in representative microscopy images. **(G)** Cathepsin B and **(H)** Cathepsin L activities, as detected by cleavage of Magic-Red substrate (red), in WT and *GBA1* heterozygous iNs. Red fluorescence from Cathepsin B/L-specific substrate cleavage is observed in representative microscopy images. In all fluorogenic plate-based activity assays, nuclei were detected by Hoechst 33258 (blue) for normalization of fluorescent signal. All lysosomal analyses were collated from 2–3 independent experiments for each of three differentiations with 10–20 wells per genotype, per experiment, on 21-day old iNs (*N* = 3, *p < 0.0001, ANOVA followed by Tukey’s *post-hoc* test).

Since GCase is a critical lysosomal enzyme, we sought to determine whether partial loss of GCase protein affects lysosome function in neurons. Using high content image analysis, we quantified the number of lysosomes per cell by identifying the organelles with the cellular stain, LysoTracker^®^. We observed that *GBA1* heterozygous-null iNs displayed a significant reduction (-50 to 70%) in the number of lysosomes (Figure 2A and Supplementary Figure S1B), while average lysosomal area of individual lysosomes remained unaffected by *GBA1* heterozygosity (Figure 2C), despite being affected by LRRK2 mutation (Schapansky et al., 2018). Since LysoTracker^®^ staining could be affected by changes in lysosomal pH, we also quantified immunofluorescently-LAMP2 stained lysosomes which would not be subject to a pH-dependent signal and observed similar data (not shown). Further, we quantified the number of lysosomes in the cell body vs. those localized within neurites and found that this decrease in lysosomal number in *GBA1* heterozygous-null iNs, is primarily due to the loss in lysosomes in the neurites (Figure 2B). To ask whether the decrease in lysosome number was due to a decrease in lysosomal biogenesis, we examined the levels of nuclear TFEB, and observed no change (Figure 2D). Interestingly, we also found no change in levels of the lysosomal protein LAMP1 and LAMP2 (Figure 2D and Supplementary Figures S1C,D). However, biochemical analyses revealed that LAMP1 and LAMP2 from *GBA1* heterozygous-null cells migrated more quickly on an SDS-PAGE than from control cells (Figure 2D and Supplementary Figures S1C,D). This observation suggested a differential post-translational modification, such as glycosylation, that is known to alter migration of proteins on SDS-PAGE (Quiza et al., 1997; Unal et al., 2008). Next, we quantified lysosomal pH using LysoSensor^TM^, a ratiometric pH-sensitive dye. We observed significant alkalinization of the lysosomes in *GBA1* heterozygous-null iNs when compared to their isogenic controls (Figure 2E), further indicating a dysfunctional endo-lysosomal pathway. This led us to ask whether this alkalinization event was sufficient to alter lysosomal protease activity, which is known to be pH sensitive. We examined general protease activity in the lysosome using a DQ-BSA conjugate dye, where BSA is fused to a green fluorescent dye such that its fluorescence is auto-quenched. Upon exposure to active proteases, the conjugate is cleaved from the fluorescent peptide fragments that freely diffuse and are thus unquenched. Using this assay, we found that lysosome protease activity was similar in WT and *GBA1* heterozygous-null neurons (Figure 2F). Given that there are many individual lysosomal enzymes that contribute to this pooled activity, we then conducted specific enzyme activity assays that are amenable to iN culture. Data showed that both clones of *GBA1* heterozygous-null neurons manifested a significant decrease in Cathepsin B (Figure 2G) and Cathepsin L activities (Figure 2H). However, given the DQ-BSA data it is likely that other enzymes are unaffected by *GBA1* heterozygosity. Additionally, we observed identical results in two independent clones of *GBA1* heterozygous-null iNs generated from BR01 background (data not shown), suggesting robust reproducibility across different iPSC lines.

### *GBA1* Heterozygous-Null Neurons Accumulate Soluble and Insoluble αSyn and Secrete Oligomeric αSyn

**FIGURE 3:**
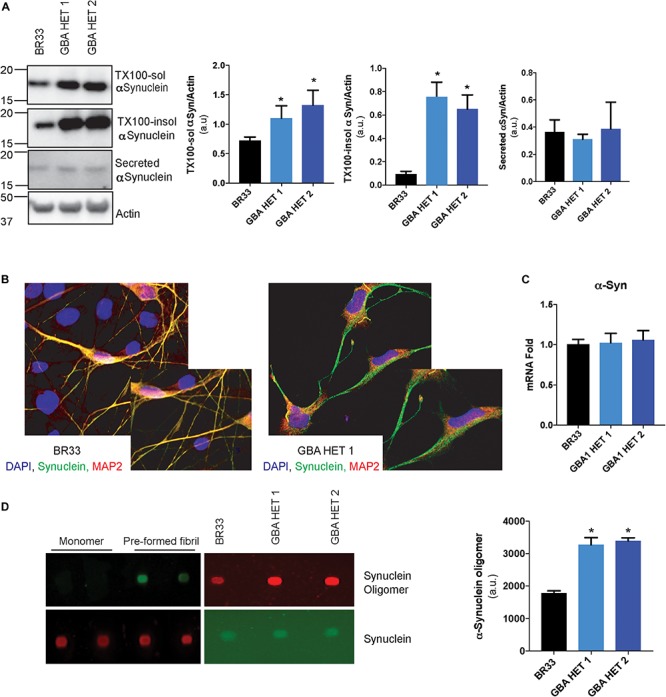
*GBA1* heterozygous-null neurons present with an accumulation of soluble and insoluble αSyn. **(A)** Western blot analysis of sequentially extracted αSyn in 0.1% triton X-100 (TX-100) followed by SDS, display protein levels of TX-100 soluble (TX-100 sol) and insoluble (TX-100 insol) αSyn in WT and *GBA1* heterozygous-null iNs. Secreted αSyn in the conditioned media is also detected by Western blot. **(B)** WT and *GBA1* heterozygous iNs were analyzed by immunofluorescence assay detecting αSyn (green). Nuclei were counterstained with DAPI (blue). **(C)** Quantitative PCR analysis of *SNCA* mRNA in WT and *GBA1* heterozygous iNs. **(D)** Dot blot with an antibody specific for oligomeric αSyn species of conditioned media from WT and *GBA1* heterozygous iNs. Oligomeric αSyn levels were quantified by normalization to total levels of secreted αSyn. All analyses were collated from three independent experiments for each of three differentiations on 21-day old iNs (*N* = 3, *p < 0.001).

Neuronal accumulation of insoluble αSyn is believed to be a key determinant of most forms of PD. Multiple lines of evidence implicate GCase loss-of-function in αSyn accumulation, and thus to PD pathogenesis. To analyze αSyn metabolism, we sequentially extracted total cellular proteins from iNs and determined the levels of detergent-soluble and insoluble αSyn. Similar to previous studies, we observed an accumulation of both soluble and insoluble forms of αSyn in *GBA1* heterozygous-null neurons of BR33 (Figure 3A) and BR01 background (data not shown), suggesting that these neurons have a decreased capacity to degrade αSyn. Increased levels of αSyn are readily visible by immunofluorescence of αSyn in *GBA1* heterozygous-null neurons, particularly within *GBA1* heterozygous-null neurites, compared to WT neurons (Figure 3B). Additionally, the accumulation of αSyn within *GBA1* heterozygous-null neurons and their processes is not due to increased neuronal maturity of the *GBA1* heterozygous-null iNs. On the contrary, these neurons have decreased neurite outgrowth (Figure 1D) but increased αSyn intensity. Critically, αSyn transcription is unchanged by *GBA1* heterozygosity (Figure 3C), indicating a protein clearance defect. Among many views on disease progression, it is also believed that αSyn may manifest with prion-like properties and the cell-to-cell transfer of extracellular αSyn may be a mechanism of spread of Lewy bodies across the brain (Danzer et al., 2012; Volpicelli-Daley et al., 2014). To consider this, we analyzed αSyn levels in conditioned media and observed no significant difference in secretion of total αSyn by *GBA1* heterozygous-null iNs (Figure 3A). Next, we sought to determine the levels of oligomeric species of secreted αSyn by using an antibody that specifically recognizes αSyn oligomers. To test the specificity of this antibody, we conducted a dot blot with purified αSyn monomer and pre-formed fibrils and observed that the antibody is unable to detect monomeric αSyn while it robustly recognized fibrillar αSyn, consistent with prior efforts validating this reagent (Lassen et al., 2018; Krashia et al., 2019; Matsui et al., 2019). Finally, we analyzed the conditioned media from WT and *GBA1* heterozygous-null iNs and found that secreted αSyn from *GBA1* heterozygous-null iNs contain more oligomeric αSyn than that from WT cells (Figure 3D). These data suggest that *GBA1* heterozygosity provokes insufficient αSyn degradation, leading to both accumulation and secretion of insoluble oligomeric species.

### *GBA1* Heterozygosity Does Not Affect Endogenous Wild-Type LRRK2 Kinase Activity

**FIGURE 4:**
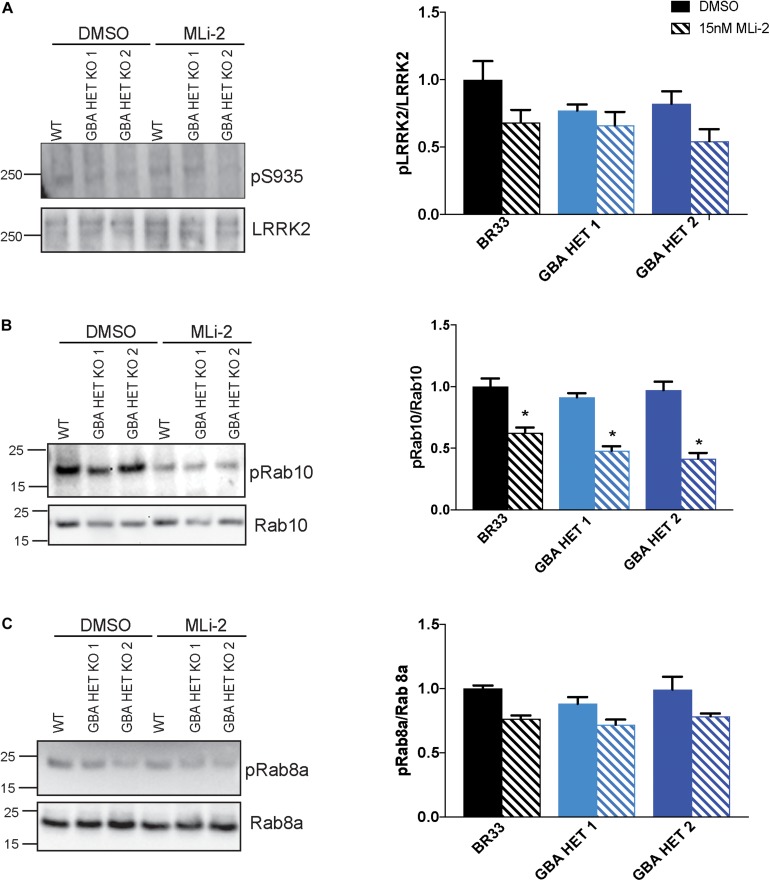
*GBA1* heterozygosity does not affect endogenous wild-type LRRK2 kinase activity. **(A)** Examination of LRRK2 phosphorylation at S935 using Western blot of WT and *GBA1* heterozygous iNs. Phosphorylation of **(B)** Rab10 and **(C)** Rab8a, putative LRRK2 kinase substrates, in WT and *GBA1* heterozygous iNs was detected by Western blot using phospho-Rab10/8a specific antibodies. Here, cells were treated with 15 nM MLi-2 for 30 min. Images were quantified by normalization to total LRRK2, Rab10, and Rab8a protein levels. All analyses were collated from three independent experiments for each of three differentiations on 21-day old iNs (*N* = 3, *p < 0.001).

We reported evidence of a crosstalk between GCase and LRRK2 kinase activity in murine astrocytes (Sanyal et al., 2020). Here, we asked whether an interaction of GCase and LRRK2 is observed in this human iPSC derived neurons. We examined multiple markers of LRRK2 activity in GCase-deficient neurons by analyzing phosphorylation status of LRRK2 S935, an indirect marker of LRRK2 activity. We also investigated the LRRK2 substrates, Rab10 and Rab8a. We observed no change in the phosphorylation levels of any of these markers in *GBA1* heterozygous-null iNs (Figures 4A–C). In addition, the total protein levels of LRRK2, Rab10 and Rab8a were unchanged across genotypes. As expected, long-term MLi-2 treatment decreased the phosphorylation of the LRRK2 substrate Rab10, confirming the efficacy of the inhibitor. A decrease in Rab8a phosphorylation was not detected, possibly due to the known poor specificity of this phospho-Rab antibody and its ability to detect Rab proteins that are not substrates of LRRK2 kinase activity (Lis et al., 2018).

### Inhibition of LRRK2 Kinase Activity Does Not Improve αSyn Metabolism in *GBA1* Heterozygous-Null Neurons

**FIGURE 5:**
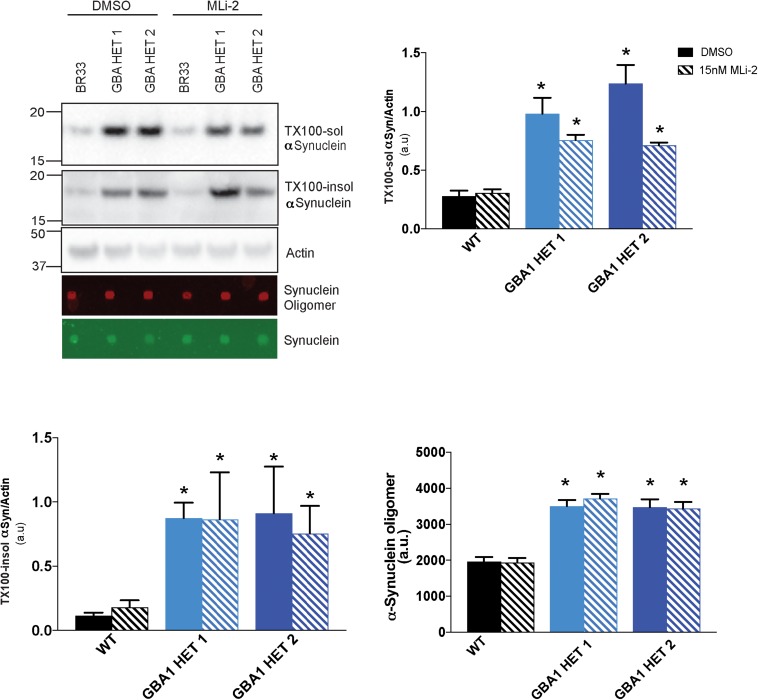
Inhibition of LRRK2 kinase activity does not reduce αSyn accumulation. Western blot analysis of sequentially extracted αSyn in 0.1% triton X-100 (TX-100) display protein levels of TX-100 soluble (TX-100 sol), insoluble (TX-100 insol) αSyn and dot blot analysis of secreted αSyn in conditioned media detect oligomeric or total αSyn in WT and *GBA1* heterozygous-null iNs treated with DMSO or 15nM MLi-2 for 14 days. All analyses were collated from three independent experiments for each of three differentiations on 21-day old iNs (*N* = 3, *p < 0.001).

Prior work from our group demonstrated that small molecule inhibitors of LRRK2 kinase activity increased the metabolism of αSyn in LRRK2 G2019S neurons (Schapansky et al., 2018). Given the cross-talk between LRRK2 and *GBA1*, and a recent report (Ysselstein et al., 2019), we analyzed whether inhibition of LRRK2 kinase activity affects *GBA1*-induced defects in αSyn metabolism. We treated WT and *GBA1* heterozygous-null iNs with the LRRK2-kinase inhibitor, MLi-2, at sub-nanomolar concentrations, for 14 days and observed no rescue of the accumulation of insoluble αSyn (Figure 5), while the levels of soluble αSyn trended toward a correction. Additionally, we also examined the levels of oligomeric αSyn secreted in the conditioned media and found no evidence for correction in the presence of MLi-2 (Figure 5).

### *GBA1*-Induced Lysosomal Perturbations Are Normalized by LRRK2 Kinase Inhibition

**FIGURE 6:**
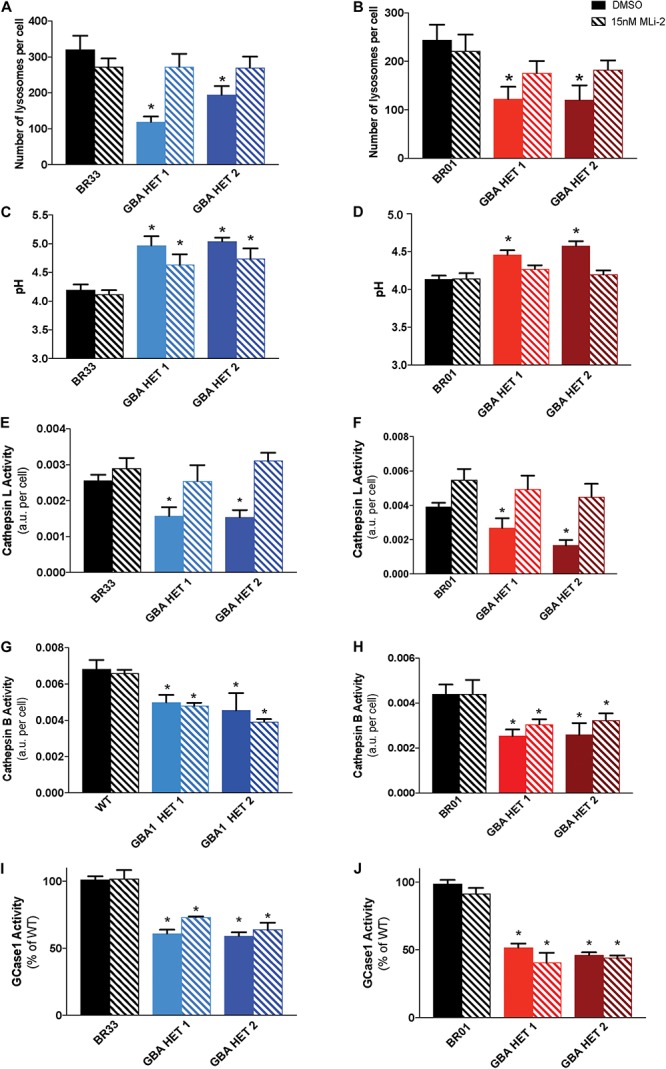
Inhibition of LRRK2 kinase activity normalizes *GBA1*-induced lysosomal perturbations. Lysosomal number, as detected by Lysotracker^TM^ staining, in WT and *GBA1* heterozygous-null iNs in **(A)** BR33 and **(B)** BR01 backgrounds. Lysosomal pH, as measured by LysoSensor^TM^, in WT and *GBA1* heterozygous iNs in **(C)** BR33 and **(D)** BR01. Cells were treated with 15 nM MLi-2 for 3 days. Cathepsin L activity, as detected by enzyme specific Magic-Red dye cleavage, in WT and *GBA1* heterozygous iNs in **(E)** BR33 and **(F)** BR01 backgrounds. Cathepsin B activity, as detected by enzyme specific Magic-Red dye cleavage, in WT and *GBA1* heterozygous iNs in **(G)** BR33 and **(H)** BR01 backgrounds. GCase activity, as detected by GCase-specific fluorogenic substrate, was analyzed following MLi-2 (15 nM) treatment for 7 days in **(I)** BR33 and **(J)** BR01 (*N* = 3). All lysosomal analyses were collated from three independent experiments with 10 wells per genotype, per experiment, for each of three differentiations on 21-days old iNs (*N* = 3, *p < 0.0001, ANOVA followed by Tukey’s *post-hoc* test).

Prior work demonstrated that reductions in WT LRRK2 kinase activity via small molecule inhibitors reversed both cytokine and lysosomal deficits induced by heterozygous *GBA1* mutation in astrocytes (Sanyal et al., 2020). Given the failure of LRRK2 inhibition to rescue changes in αSyn metabolism, we sought to determine whether the underlying lysosomal dysfunction was broadly rescued by LRRK2 inhibition, and if not, whether selective deficits were corrected while others were not. Data showed that MLi-2 treatment resulted in a near-complete rescue of the *GBA1*-induced decrease in lysosomal number in both isogenic clones, from two independent WT backgrounds (BR33 and BR01) (Figures 6A,B). In addition, the lysosomes were partially re-acidified by LRRK2 inhibitor treatment in the *GBA1* heterozygous-null iNs. While GBA1/BR33 heterozygous-null iNs trended toward decreased pH (Figure 6C), GBA1/BR01 iNs had significantly re-acidified lysosomes (Figure 6D). Individual lysosome area was not affected by *GBA1* heterozygosity, nor was it influenced by LRRK2 kinase inhibition (data not shown). We also analyzed the effect of LRRK2 kinase inhibition on lysosomal proteases. Data in BR33 and BR01 mutant lines revealed that Cathepsin L activity was normalized by LRRK2 inhibition (Figures 6E,F). Interestingly, Cathepsin B activity was not corrected irrespective of the recovered Cathepsin L activity and the rescue of broader lysosomal properties (Figures 6G,H). It is interesting to note that in our recent work in murine neurons, Cathepsin B-like activity was inversely correlated with αSyn levels (Schapansky et al., 2018), as it was here in human neurons. Lastly, we asked whether LRRK2 kinase inhibition affected GCase activity in either WT or *GBA1* heterozygous-null neurons. Our data showed no change in GCase activity upon 7 day (Figures 6I,J), or 3 day (data not shown) treatment with MLi-2 in any of the iPSC derived neurons. These data suggest that while LRRK2 impinges on pathways downstream of GCase deficiency, WT LRRK2 activity does not directly affect GCase activity in these cells.

## Discussion

The ability to use human iPSCs to model neurological disorders has proven to be a potent and meaningful tool to better understand molecular mechanisms that are altered in these uniquely human diseases. iPSCs can be derived directly from patients or genetically manipulated to mirror a disease state, thus making it possible to study human neurons, an otherwise inaccessible cell type. In this study we sought to model the reductions in GCase activity that impart substantially elevated risk of PD through inheritance of heterozygous loss-of-function mutations in *GBA1*. To date, the impact of pure, heterozygous loss-of-function in the absence of a mutated missense *GBA1* mutation has not been explored. To address this, we used CRISPR/Cas9 genome editing to induce *GBA1* heterozygosity by a targeted allelic loss of *GBA1* in healthy control human iPSCs (BR33 and BR01). These cells and their isogenic controls were then differentiated to cortical neuronal fate, given the unique prevalence of dementia in GBA1-PD (Liu et al., 2016) and the prevalence of αSyn throughout both cortical and sub-cortical brain regions in PD (Hurtig et al., 2000; Jellinger, 2012). Human iPSC-derived neurons partially deficient in GCase manifested with broad lysosomal defects including decreases in lysosome number and alkalinization of lysosomal pH. These cells displayed decreased lysosomal Cathepsin B and L activities, as compared to their isogenic controls. Lysosomal function was altered similarly in iNs generated from both BR33 and BR01 lines and across multiple clones, suggesting that these observations are robustly reproducible across different genomic backgrounds and are not confounded by variabilities induced by genome targeting, clonal selection, reprogramming or differentiation.

Multiple lines of evidence support the cell-to-cell transfer of insoluble αSyn (Braak et al., 2003; Danzer et al., 2012; Luk et al., 2012; Volpicelli-Daley et al., 2014; Jones et al., 2015). Additionally, GCase-null mice were found to exhibit an accumulation of endogenous αSyn and the formation of its insoluble oligomers (Mazzulli et al., 2011; Sardi et al., 2011). Consistent with observations across multiple *GBA1* model systems (Mazzulli et al., 2011; Woodard et al., 2014; BR1, 2018; Kim et al., 2018; Maor et al., 2019), we found an accumulation of both soluble and insoluble αSyn in human heterozygous-null *GBA1* neurons with no change in its transcription levels. Interestingly, although the total levels of secreted αSyn remain unaffected by GCase deficiency, αSyn oligomers were selectively enriched in the conditioned media of *GBA1* heterozygous-null neurons, when compared to their isogenic wild-type control. These data might suggest a greater propensity for the spread of αSyn pathology, but future work in animal models will be best suited to fully address the implications of this altered αSyn release. Both lysosomal deficiency and αSyn accumulation can contribute to decreased neuronal maturation in primary rodent neurons and neuronal cell culture model (Ramonet et al., 2011; Koch et al., 2015; Wrasidlo et al., 2016; Prots et al., 2018; Srikanth et al., 2018). Accordingly, we observed that *GBA1* heterozygous-null iNs display a decrease in neurite length, as well as number of neurite branch points. Further work will be required to dissect the mechanisms underlying this novel phenotype.

Several autosomal dominant missense mutations in LRRK2 are causal for PD and aberrant LRRK2 activity can influence both lysosomal dysfunction and αSyn dyshomeostasis (Henry et al., 2015; Hockey et al., 2015; Bae et al., 2018; Novello et al., 2018; Schapansky et al., 2018). We and others have shown altered lysosomal morphology and decreased lysosomal proteins in LRRK2 G2019S knock-in mice and in primary cultured neurons (Herzig et al., 2011; Hockey et al., 2015; Kuwahara et al., 2016; Schapansky et al., 2018; Wallings et al., 2019). These neurons also showed alkalinized lysosomes and the accumulation of insoluble αSyn, as was the case in GCase-deficient neurons in the present study. Importantly, we have observed rescue of the lysosomal defects by LRRK2 kinase inhibition both in LRRK2 mutant neurons (Schapansky et al., 2018) and *GBA1* mutant astrocytes (Sanyal et al., 2020). Here, our data revealed that although endogenous LRRK2 kinase activity was not affected by *GBA1* heterozygosity, inhibition by exogenous means (MLi-2) led to the normalization of lysosomal number, pH, and Cathepsin L activity. Results indicate that upon treatment with LRRK2 inhibitor, MLi-2, partial re-acidification of lysosomal pH was observed in GBA1/BR33 heterozygous neurons while lysosomal pH in GBA1/BR01 heterozygous neurons was completely corrected, potentially highlighting the role of patient-to-patient variability commonly observed in disease pathogenesis. Furthermore, we observed that four clones of *GBA1* heterozygous iNs that were generated from two independent iPSC backgrounds displayed normalization of lysosomal number and correction of Cathepsin L activity by LRRK2 inhibition. Importantly, Cathepsin B activity was not normalized by LRRK2 inhibition. We have previously demonstrated that Cathepsin B is important for the degradation of αSyn in neurons (Schapansky et al., 2018), consistent with work from another group (Tsujimura et al., 2015). Accordingly, we observed here that LRRK2 inhibition was unable to normalize the increased levels of αSyn in *GBA1* heterozygous-null iNs. Given the changes in oligomeric αSyn in the *GBA1* heterozygous-null iNs and the lack of effect of LRRK2 inhibition on αSyn and Cathepsin B, we hypothesize that Cathepsin B is essential for the degradation of intracellular αSyn in human neurons.

Broad lysosomal deficits and their normalization by LRRK2 kinase inhibition were observed both in *GBA1* heterozygous *D409V* knockin murine astrocytes (Sanyal et al., 2020) and in *GBA1* heterozygous-null human iNs (this study). However, several differences were also noted, possibly as a manifestation of cell-type specific differences or differences occurring from missense mutation vs. allelic loss. The number of lysosomes was decreased ~50% in both cell types, however, the extent of lysosomal alkalinization was greater in iNs (pH∼1 unit) than in astrocytes (pH∼0.5 units. Cathepsin B activity was inhibited in iNs (-30%) to a greater extent than in astrocytes (-20%). While Cathepsin L activity was unaffected in astrocytes, it was significantly decreased in iNs (-40%). The effect of LRRK2 inhibition also exhibited cell-type specific differences. Upon inhibitor treatment, lysosomal pH was normalized in astrocytes but not lysosomal number. In iNs, both the decrease in lysosomal number and alkalinization of lysosomes were normalized. Furthermore, the decrease in Cathepsin B activity, which was rescued in murine astrocytes, remain unchanged by LRRK2 inhibitor in *GBA1* heterozygous iNs. Therefore, the cell-type specific changes arising from GCase deficiency, and the effects of LRRK2 kinase inhibition in GBA-PD models are quite complex. Importantly, we observed that MLi-2 did not significantly impact lysosomal functions in WT astrocytes or neurons, indicating another layer of specificity in terms of drug responsiveness in cells. This observation is particularly important since in a recent study MLi-2 at 600 nM was shown to indiscriminately increase GCase activity in WT, GBA1 mutant and LRRK2 mutant neurons (Ysselstein et al., 2019). In contrast to those data, we observed no rescue of GCase activity in any cells tested following treatment with a concentration of 15 nM, where this lower concentration is roughly 10-fold greater than the IC_50_ (1.4 nM).

LAMP1 and LAMP2 are trafficked to the lysosome via the ER-trans golgi network where they are differentially glycosylated (Carlsson and Fukuda, 1992). Consequently, different glycosylated forms of LAMP1 and LAMP2 have unique migration rates on an SDS-PAGE. We have reproducibly observed the accumulation of a faster migrating species of LAMP1 and LAMP2 in *GBA1* heterozygous-null neurons, indicating an irregular glycosylation of these proteins as a function of *GBA1* deficiency. These data are consistent with ER stress reported by others in cells expressing *GBA1* mutations (Fernandes et al., 2016; Sanchez-Martinez et al., 2016) and importantly indicate that these effects are not limited to conditions where cells express a mis-folded, mutant *GBA1* protein but rather are more directly associated with reduced GCase activity in the cell. The improper trafficking of key lysosomal proteins may contribute to the lysosomal alkalinization, or other deficiencies, we found in this study. Our recent data in *GBA1* heterozygous *D409V* knockin astrocytes also revealed alkalinization of lysosomes, highly consistent with the effects of pure GCase deficiency seen here. Key players that coordinate trafficking of lysosomal proteins belong to the Rab family of small GTPase (Cantalupo et al., 2001; del Toro et al., 2009; Chen et al., 2010). Recent studies have shown that several Rab GTPases are implicated in PD progression as they are phosphorylated and thought to be inactivated by LRRK2 (Steger et al., 2016). Thus, it is highly relevant that *GBA1* mutant lysosomes could be re-acidified by a LRRK2 inhibitor. Our observation of glycosylation defects and lysosomal alkalinization in *GBA1*-deficient neurons underscores the potentially broader requirement of proper GCase activity in Rab-dependent trafficking.

Collectively, our findings suggest that inhibition of LRRK2 kinase activity may be sufficient to exert therapeutically relevant effects in neurons and astrocytes in the context of GBA-PD models, but there are also limitations. Our data also indicate a critical role for physiological GCase activity in cellular trafficking and is not restricted to its known function in the lysosome. Finally, LRRK2-GCase interactions not only reveal critical aspects of endogenous LRRK2 signaling but also provide evidence for a functional biochemical intersection between signaling cascades regulated by these two proteins that converge to influence the lysosome.

## Data Availability Statement

All datasets generated for this study are included in the article/Supplementary Material.

## Author Contributions

ML and AS designed the study and wrote the manuscript. AS and HN conducted the experiments and analyzed the data. EG standardized αSyn oligomer detection. SL executed Sanger sequencing of iPSC clones. ML supervised the entire study. All authors revised and agreed to the final version of the manuscript.

## Conflict of Interest

The authors declare that the research was conducted in the absence of any commercial or financial relationships that could be construed as a potential conflict of interest.

## Footnotes

1 http://crispr.mit.edu

## Supplementary Material

FIGURE S1 **(A)** Sanger sequencing of *GBA1* heterozygous-null clones in WT iPSC backgrounds BR01 and BR33. **(B)** 63X magnification images of LAMP2 (green) stained lysosomes in BR33 (WT) and GBA HET1/BR33 neurons. Nuclei were stained with DAPI (blue). **(C)** Western blot analysis of LAMP2 in WT (BR33) and *GBA1* heterozygous iNs (GBA HET 1 and GBA HET 2) neurons **(D)** Western blot analysis of six biological replicates of WT (BR33) and *GBA1* heterozygous iNs (GBA HET 1 and GBA HET 2) neurons, detecting glycosylated species of LAMP1. Quantification of LAMP1 was normalized to loading control Actin.
