# Supplementary figures and images for: LRRK2 Kinase Inhibition Rescues Deficits in Lysosome Function Due to Heterozygous GBA1 Expression in Human iPSC-Derived Neurons
Source: Front Neurosci. 2020 May 15;14:442. doi: 10.3389/fnins.2020.00442 (PMC7243441; doi:10.3389/fnins.2020.00442)

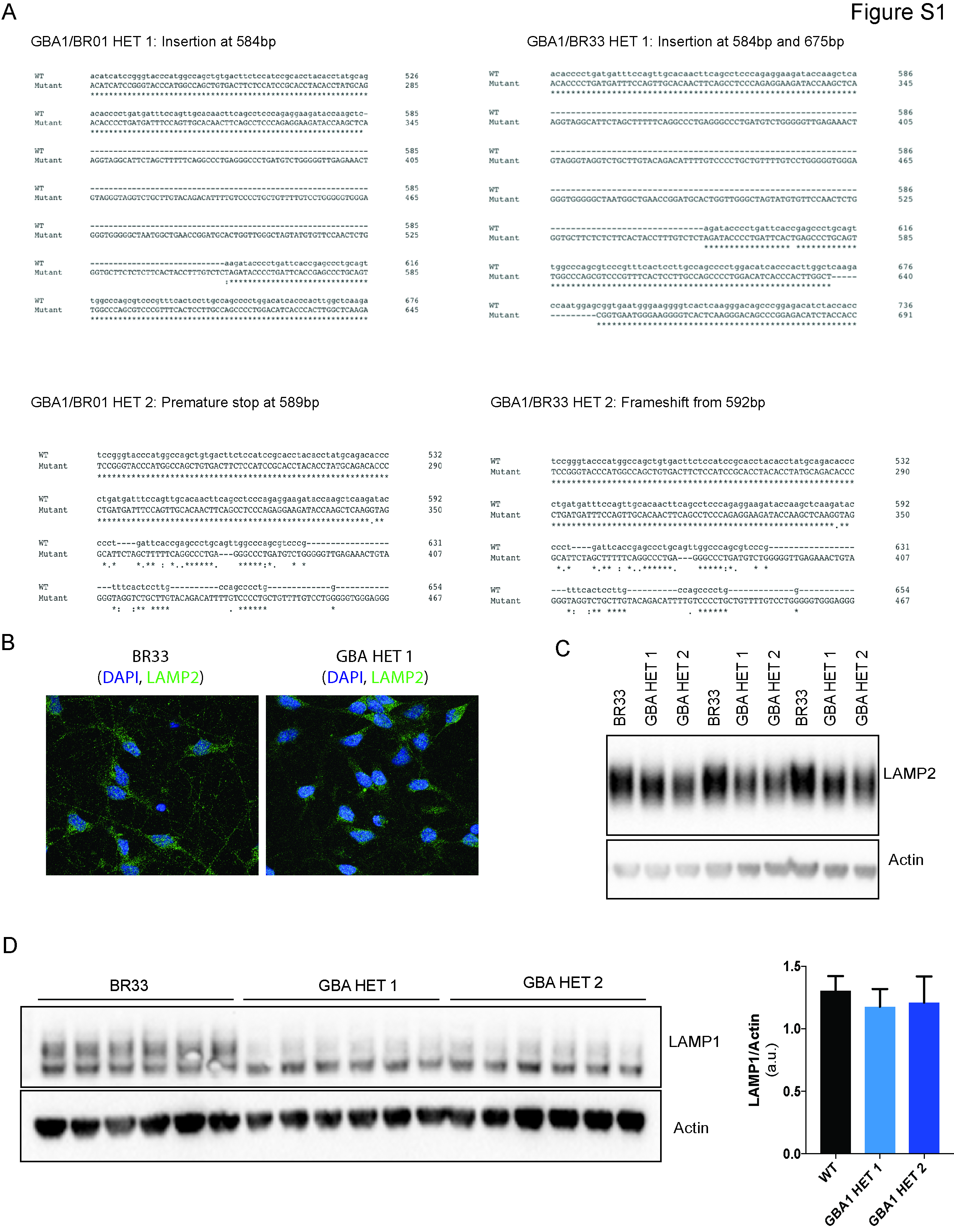

Supplement: FIGURE S1 — (A) Sanger sequencing of GBA1 heterozygous-null clones in WT iPSC backgrounds BR01 and BR33. (B) 63X magnification images of LAMP2 (green) stained lysosomes in BR33 (WT) and GBA HET1/BR33 neurons. Nuclei were stained with DAPI (blue). (C) Western blot analysis of LAMP2 in WT (BR33) and GBA1 heterozygous iNs (GBA HET 1 and GBA HET 2) neurons (D) Western blot analysis of six biological replicates of WT (BR33) and GBA1 heterozygous iNs (GBA HET 1 and GBA HET 2) neurons, detecting glycosylated species of LAMP1. Quantification of LAMP1 was normalized to loading control Actin. [file Image_1.TIF]
